# Activation of p53/miR-34a Tumor Suppressor Axis by Chinese Herbal Formula JP-1 in A549 Lung Adenocarcinoma Cells

**DOI:** 10.1155/2016/5989681

**Published:** 2016-12-18

**Authors:** Chih-Jung Yao, Jyh-Ming Chow, Pei-Chun Lin, Tsai-Shu Hu, Hui-Ching Kuo, Jhy-Shrian Huang, Kuan-Jen Bai, Gi-Ming Lai

**Affiliations:** ^1^Cancer Center, Wan Fang Hospital, Taipei Medical University, Taipei 11696, Taiwan; ^2^Comprehensive Cancer Center of Taipei Medical University, Taipei 11031, Taiwan; ^3^Department of Internal Medicine, School of Medicine, College of Medicine, Taipei Medical University, Taipei 11031, Taiwan; ^4^Division of Hematology and Medical Oncology, Department of Internal Medicine, Wan Fang Hospital, Taipei Medical University, Taipei 11696, Taiwan; ^5^Division of Pulmonary Medicine, Department of Internal Medicine, Wan Fang Hospital, Taipei Medical University, Taipei 11696, Taiwan; ^6^School of Respiratory Therapy, College of Medicine, Taipei Medical University, Taipei 11031, Taiwan

## Abstract

Lung cancer is the leading cause of cancer death worldwide; the most common pathologic type is lung adenocarcinoma (LADC). In spite of the recent progress in targeted therapy, most LADC patients eventually expired due to the inevitable recurrence and drug resistance. New complementary agent with evidence-based molecular mechanism is urgently needed. MiR-34a is an important p53 downstream tumor suppressor, which regulates apoptosis, cell-cycle, EMT (epithelial mesenchymal transition), and so forth. Its expression is deficient in many types of cancers including LADC. Here, we show that a Chinese herbal formula JP-1 activates p53/miR-34a axis in A549 human LADC cells (p53 wild-type). Treatment with JP-1 induces p53 and its downstream p21 and BAX proteins as well as the miR-34a, resulting in growth inhibition, colony formation reduction, migration repression, and apoptosis induction. Accordingly, the decreases of miR-34a downstream targets such as CDK6, SIRT1, c-Myc, survivin, Snail, and AXL were observed. Moreover, JP-1 activates AMPK*α* and reduces mTOR activity, implying its inhibitory effect on the energy-sensitive protein synthesis and cell proliferation signaling. Our results show that JP-1 activates p53/miR-34a tumor suppressor axis and decreases proteins related to proliferation, apoptosis resistance, and metastasis, suggesting its potential as a complementary medicine for LADC treatment.

## 1. Introduction

Lung cancer is the most common cancer and remains the leading cause of cancer-related mortality worldwide [1, 2]. The most commonly diagnosed type is lung adenocarcinoma (LADC), which has a poor prognosis [3]. Even though various methods for diagnosis and treatment have been improved in recent decades, the 5-year survival rate of LADC is still unsatisfied [4]. There is a pressing need for new approaches to tackle this disease. In addition to the development of synthetic cytotoxic compounds or tyrosine kinase inhibitors, patients resort to complementary and alternative medicines for improving clinical outcomes. In this regard, Chinese herbal medicine with anticancer activities and low toxicity represents a viable resource and potential candidate.

The powerful tumor suppressor p53 is a transcription factor, which plays a crucial role in the regulation of cell-cycle, apoptosis, DNA repair, senescence, and angiogenesis [5]. Although the function of p53 is impaired in approximately half of human cancers by deletion or mutation of the gene encoding p53 protein, TP53 [5], it is still regarded as an important therapeutic target in oncology [6]. In the remaining 50% of human cancers with wild-type p53 status, blocking the negative regulation of p53 by MDM2 (human murine double minute 2) has become a promising cancer therapeutic strategy [5, 6]. A variety of small molecule or peptidic compounds had been developed to inhibit MDM2 [7]. On the other hand, many herbal extracts have been shown to induce growth arrest or apoptosis of cancer cells via p53 activation [8–13]. Thus, the Chinese herbal medicine represents a potential resource for searching for proper agent to restore p53 function of cancer cells.

JP-1 is a Chinese herbal formula developed by Dr. Peter Sheng, a medical oncologist in Cincinnati, Ohio, USA, which mainly consists of* Ganoderma lucidum*,* Herba Scutellaria barbata*,* Scutellaria baicalensis*,* Oldenlandia diffusa*,* Astragalus membranaceus*,* Codonopsis Pilosula*, and* Bulbus fritillariae cirrhosae*, and so forth. Based on Dr. Sheng's clinical observation (unpublished data), JP-1 showed the effects to stabilize the tumor progression of chemotherapy-refractory patients with LADC and improved their quality of life, implying its potential as a complementary medicine. However, its mechanism of action has not yet been investigated. We attempt to explore the effects of JP-1 on LADC cells to evaluate its anticancer activities and elucidate the underlying molecular mechanisms.

Activation of p53 leads to transactivation of its target genes, like p21, BAX, Fas, and so forth, leading to growth arrest or apoptosis of cancer cells [14]. Recently, several studies have revealed microRNA (miR) components in the p53 tumor suppressor network [15]. MiRs are a recently identified large family of 21–25-nucleotide noncoding RNAs that regulate gene expression by targeting mRNA in a sequence-specific manner, resulting in either reduced translation efficiency or cleavage of the target mRNAs [16]. In recent years, miRs have received great attention in the research of LADC. Many miRs are misregulated in LADC and expected to play critical roles in the proliferation, metastasis, and chemoresistance [17, 18]. MiR-34a, a bona fide p53 transcriptional target, has been shown to inhibit genes, such as CDK6, SIRT1, c-Myc, survivin, Snail, and AXL, involved in cell-cycle progression, apoptosis resistance and epithelial mesenchymal transition (EMT), and so forth, [19–21]. The expression of miR-34a is downregulated in many human cancers including lung cancer [22] and restoration of miR-34a results in tumor repression in mouse models of LADC [23, 24]. These observations imply that modulation of miR-34a expression might be a potential novel strategy to treat LADC.

In an attempt to investigate the molecular mechanism underlying JP-1-mediated anticancer activities, we explored its effects on the p53/miR-34a tumor suppressor axis in A549 human LADC cells harboring wild-type p53 gene. Our results show that JP-1 upregulates p53 protein level to increase those of p21 and BAX, resulting in growth arrest and apoptosis of A549 cells. Of note, the transcription of miR-34a is activated by JP-1 and the downstream targets of miR-34a such as CDK6, SIRT1, c-Myc, survivin, Snail, and AXL are repressed accordingly. Our findings suggest the effects of JP-1 on the activation of p53/miR-34a tumor suppressor axis, which may explain the clinical benefit observed in JP-1-treated patients.

## 2. Materials and Methods

### 2.1. Cell Culture

The A549 human lung adenocarcinoma cells and HS68 primary human fetal foreskin fibroblast cells were purchased from the American Type Culture Collection (Manassas, VA, USA). A549 cells were maintained in RPMI1640 and HS68 cells were in DMEM. Both mediums were supplemented with 10% fetal bovine serum (Caisson, North Logan, UT, USA), 1x penicillin-streptomycin-glutamine (Corning, Manassas, VA, USA), and 1x nonessential amino acids (Corning, Manassas, VA, USA). Cells were cultured at 37°C in a water-jacketed 5% CO_2_ incubator.

### 2.2. Preparation of JP-1

The original JP-1 powder was provided by Dr. Peter Sheng, a medical oncologist in Cincinnati, Ohio, USA. It was extracted by 38% ethanol. After lyophilization, the stock solution of JP-1 dissolved in ethanol at concentration 100 mg/mL was prepared. It was diluted in sterile culture medium immediately prior to use. The final concentrations of ethanol were all below 0.1%.

### 2.3. Cell Viability Assay by Sulforhodamine B (SRB) Staining

A549 cells were seeded in a 96-well plate at a density of 1.5 × 10^3^ cells/well in 10% FBS-RPMI medium. HS68 cells were seeded in a 96-well plate at a density of 8 × 10^3^ cells/well in 10% FBS-DMEM medium. After 24 h of incubation, cells were treated with various doses of JP-1 for another 72 h. Cells were then harvested and fixed by 10% trichloroacetic acid (TCA). After fixing, cells were washed by distilled water and stained viable cells by 0.4% (w/v) SRB dye dissolved in 1% acetic acid. After staining for 30 min, the unbound dye was then washed away by 1% acetic acid and the plate was air-dried. The cell-bound SRB dye was then dissolved in 200 *μ*L of 10 mM Tris base and the absorbance was read on a microplate reader (BioTek ELx800, Winooski, VT, USA) at a wavelength of 570 nm. The absorbance was directly proportional to the cell number over a wide range.

### 2.4. Cell-Cycle Analysis

A total of 4 × 10^5^ A549 cells/10 cm dish were incubated for 24 h after seeding and then treated with the indicated doses of JP-1 for another 48 h. Cells treated with culture medium only were used as a control. On the day of harvest, the cells were trypsinized using Trypsin-EDTA (Invitrogen) and washed twice with ice-cold PBS and then fixed in cold 70% ethanol for overnight at 4°C. Cells were washed twice with PBS after centrifuging at 200 ×g for 10 min and incubated with 100 *μ*g/mL of PI (propidium iodide) and 100 *μ*g/mL RNAse together at 37°C for 30 min and then transferred onto ice or stored at 4°C protected from light. The percentage of cells at different phases of cell-cycle was then measured by flow cytometer (Beckman Coulter EPICS XL, Fullerton, CA, USA).

### 2.5. Colony Formation Assay

A549 cells were seeded onto 6-well pates at a density of 200 cells per well. After 24 h, cells were treated with 75 and 150 *μ*g/mL of JP-1, respectively. After 10 days of treatment, colonies were stained with Amido black and the number of colonies containing more than 50 cells was counted.

### 2.6. Semiquantitative Reverse-Transcription Polymerase Chain Reaction (RT-PCR)

A total of 4 × 10^5^ A549 cells/10 cm dish were incubated for 24 h after seeding and then treated with variable doses of JP-1 for another 48 h. Total RNA was extracted using TRIzol reagent according to the manufacturer's instructions and was reversely transcribed to cDNA by First-Strand cDNA Synthesis Kit (Fermentas, Lithuania). The PCR was carried out in a 50 *μ*L reaction mixture that contained 1 *μ*L of cDNA as template, 1 *μ*M specific oligonucleotide primer pair, and 25 *μ*L Taq mixture containing 0.5 unit of Taq DNA polymerase. The PCR primers used for amplification were as follows: miR-34a forward primer 5′-TTTCCTTCTTAT CAACAGGTGCT-3′ and miR-34a reverse primer 5′-ATCTCTCGCTTCATCTTCCCTCT-3′; U6 forward primer 5′-CTCGCTTCGGCAGCACA-3′ and U6 reverse primer 5′-AACGCTTCAC GAATTTGCGT-3′. PCR products were run on 1.5% agarose gels for identification.

### 2.7. Western Blotting

A total of 4 × 10^5^ A549 cells/10 cm dish were incubated for 24 h after seeding and then were treated with variable doses of JP-1 as indicated in figures. On the day of harvest, the whole-cell lysates were prepared with 1x radioimmunoprecipitation (RIPA) lysis buffer (Millipore, Billerica, MA, USA) containing 1x tyrosine phosphatase inhibitor cocktail (FC0020-0001, BIONOVAS, Toronto, Canada), 1x protease inhibitor cocktail-full range (FC0070-0001, BIONOVAS, Toronto, Canada), and 1x serine/threonine phosphatase inhibitor cocktail (FC0030-0001, BIONOVAS, Toronto, Canada).

Samples (10 *μ*g) of protein extract were size fractionated electrophoretically by 10% polyacrylamide SDS-PAGE gel and transferred onto a PVDF membrane using the BioRad Mini Protean electrotransfer system (CA, USA). The membranes blots were incubated with 5% milk in PBST for 1 h to block nonspecific binding and then were incubated with primary antibodies overnight at 4°C. The membranes were detected with an appropriate peroxidase-conjugated secondary antibody incubated at room temperature for 1 h. Intensive PBS washing was performed after each time of incubation. The immune complexes were visualized using an enhanced chemiluminescence detection system (ECL, Perkin Elmer, Waltham, MA, USA) according to the manufacturer's instructions. Primary antibodies against BAX (1 : 1000, ab32503), CDK6 (1 : 1000, ab124821), GAPDH (1 : 1000, ab8245), p21 (1 : 1000, ab109199), p-mTOR (phospho S2448) (1 : 1000, ab109268), and Vimentin (1 : 1000, ab92547) were purchased from Abcam (Cambridge, MA, USA); primary antibodies for cleaved PARP (1 : 1000, #5625), cleaved caspase-9 (1 : 1000, #7237), cleaved caspase-3 (1 : 1000, #9664), p-AMPK*α* (Thr172) (1 : 1000, #2535), p-pRb (1 : 1000, #9308), SIRT1 (1 : 1000, #8469), survivin (1 : 1000, #2808), and Snail (1 : 1000, #3879) were purchased from Cell Signaling (Danvers, MA, USA); Primary antibody against full length caspase-8 (1 : 1000, #1006-1) was purchased from Epitomics (Burlingame, CA, USA). Primary antibodies for c-Myc (1 : 1000, sc-40), AXL (1 : 500, sc-1096), and p53 (1 : 500, sc-98) were purchased from Santa Cruz Biotechnology (San Diego, CA, USA).

### 2.8. Wound Healing Assay

In vitro wound healing assay was performed using the IBIDI Culture-Inserts (GmbH, Munich, Germany) according to the instruction of manufacture. An IBIDI culture insert consists of two reservoirs separated by a 500 *μ*m thick wall. It was placed into the well of 12-well plate and slightly pressed on the top to ensure tight adhesion. An equal number of A549 cells (70 *μ*L; 3 × 10^5^ cells/mL) were added into the two reservoirs of the same insert. After 24 h, the insert was gently removed creating a gap of 500 *μ*m. The cells were then treated as indicated and the migration was observed and photographed after 12, 24, and 48 h by a digital microscope camera (PAXcam2+, Villa Park, IL, USA) adapted to an inverted microscope (Olympus/CKX31, Tokyo, Japan).

### 2.9. Photograph of the Cells

The phase-contrast images of cells were photographed by a digital microscope camera (PAXcam2+, Villa Park, IL, USA) adapted to an inverted microscope (Olympus/CKX31, Tokyo, Japan).

### 2.10. Statistical Analysis

**Figure 1:**
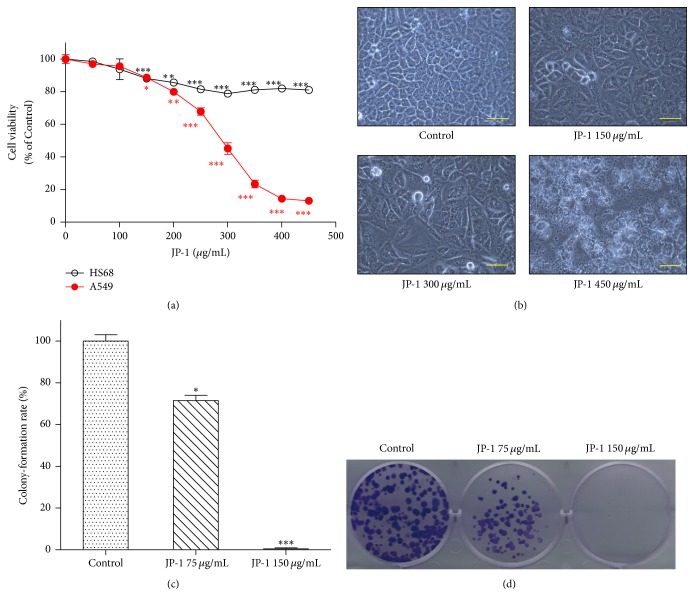
JP-1 inhibits the proliferation and colony formation of A549 cells but only slightly affects the growth of HS68 primary fibroblast cells. (a) The cell viability of A549 cells after treatment with JP-1 for 72 h was measured by SRB binding assay. Data (mean ± SE) are expressed as a percentage compared to the control. (b) After 48 h of treatment, the effect of JP-1 on A549 cells was examined by phase-contrast microscopy, scale bar = 50 *μ*m. (c) Colonies formed by control and JP-1-treated A549 cells were assayed after 10 days of incubation. (d) Representative picture of colonies formed by the cells described in (c). Cell viability and colony formation data are expressed as mean ± SE and analyzed as described in Section 2.

Cell viability and colony formation data are expressed as mean ± SE. In Figures 1(a), 1(c), and 6(c), differences between control and JP-1-treated groups were evaluated by one-way ANOVA followed by Dunnett's *t*-test. Significance values are represented by single (*p* < 0.05), double (*p* < 0.01), and triple (*p* < 0.001) asterisks.

## 3. Results

### 3.1. JP-1 Inhibits the Proliferation and Colony Formation of A549 Cells

Firstly, we tested the effects of JP-1 on the proliferation and colony formation of A549 human LADC cells. The result of SRB staining showed that treatment with JP-1 for 72 h significantly decreased the growth of A549 cells in a dose-dependent manner (Figure 1(a)). At doses of 300 and 450 *μ*g/mL, JP-1 reduced the A549 cell viability to 45.1% and 13% of control, respectively (Figure 1(a)). By contrast, the same doses of JP-1 only slightly reduced the cell viability of HS68 primary human fetal foreskin fibroblast cells to 78.7% and 81% of control (Figure 1(a)), respectively, implying the preferential inhibition of cancer cells by JP-1. After treatment with JP-1 (300 *μ*g/mL) for 48 h, significant decrease of A549 cell number accompanied with some detached cells could be observed under microscope (Figure 1(b)). When the dose was increased to 450 *μ*g/mL, marked cell death was observed (Figure 1(b)). Similar result was found in colony formation assay. At dose of 75 *μ*g/mL, JP-1 decreased the number of A549 colonies to 71.5% of control (Figures 1(c) and 1(d)). When the dose was increased to 150 *μ*g/mL, almost no colony was formed (Figures 1(c) and 1(d)).

### 3.2. JP-1 Induces Apoptotic Sub-G1 Fraction in A549 Cells

**Figure 2:**
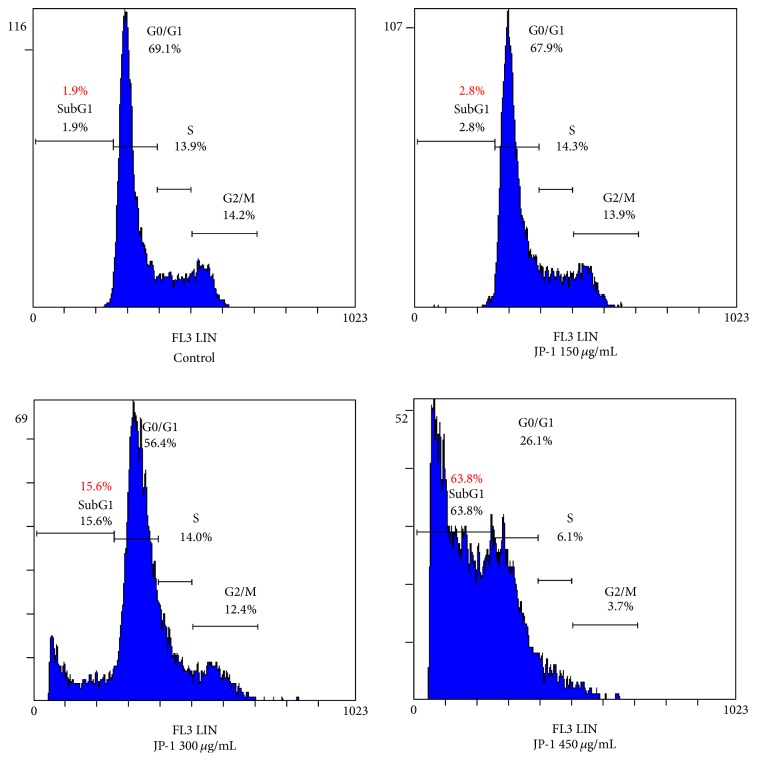
Representative flow cytometry histograms of A549 cells stained with PI after treatment with JP-1 for 48 h. JP-1 dose-dependently increased the apoptotic sub-G1 fraction in A549 cells. The percentages of cells in different phases of the cell-cycle are shown in Table 1.

After treatment with JP-1 for 48 h, the A549 cells were stained with PI and analyzed by flow cytometry to measure the change in cell-cycle distribution. As shown in Figure 2 and Table 1, JP-1 significantly increased the apoptotic sub-G1 fraction from 1.9% in control group to 15.6% and 63.8% at dose of 300 and 450 *μ*g/mL, respectively. Apoptosis induction apparently plays an important role in JP-1-mediated suppression of A549 cells and the cell-cycle might be inhibited by JP-1 via induction of the cyclin-dependent kinase (CDK) inhibitor.

### 3.3. JP-1 Decreases CDK6 Protein and Activates the AMPK Pathway in A549 Cells

**Figure 3:**
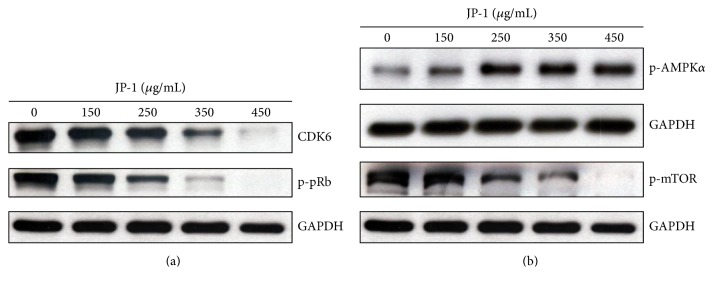
Effects of JP-1 on the AMPK/mTOR and CDK6/pRb cascades in A549 cells. (a) JP-1 decreased the protein levels of CDK6 and phosphorylated pRb (p-pRb) in a dose-dependent manner after 48 h of treatment. (b) JP-1 dose-dependently increased the phosphorylated AMPK*α* protein (p-AMPK*α*) and decreased the phosphorylated mTOR (p-mTOR) after 48 h of treatment. Cell lysates were analyzed by Western blot, using GAPDH as the loading control.

We then examined the levels of proteins that regulate cell-cycle progression and signaling transduction in JP-1-treated A549 cells. After 48 h of treatment, JP-1 dose-dependently decreased the protein levels of CDK6 (Figure 3(a)). CDK6 was known to promote the phosphorylation of tumor-suppressive retinoblastoma protein (pRb) to inactivate its function in constraining cell-cycle progression [25]. In accordance with this, the phosphorylated pRb (p-pRb) was decreased by JP-1 in a dose-dependent manner (Figure 3(a)). In the aspect of signaling pathway modulation, JP-1 activated the AMPK (AMP-activated protein kinase) signaling pathway. The phosphorylated/activated AMPK*α* protein (p-AMPK*α*) was increased after treatment with JP-1 for 48 h (Figure 3(b)). AMPK is a well-known cellular energy sensor, which can be activated by the rise of AMP/ATP ratio [26]. The activated AMPK can inhibit the activity of mTOR (mammalian target of rapamycin), which, in turn, inhibits cell proliferation and protein synthesis [27]. Accordingly, the protein level of phosphorylated/activated mTOR (p-mTOR) in JP-1-treated cells was reduced significantly in a dose-dependent manner (Figure 3(b)). JP-1 might induce metabolic stress in cancer cells, resulting in the activation of tumor suppressors.

### 3.4. JP-1 Induces p53 and Its Downstream Effectors p21 and BAX in A549 Cells

**Figure 4:**
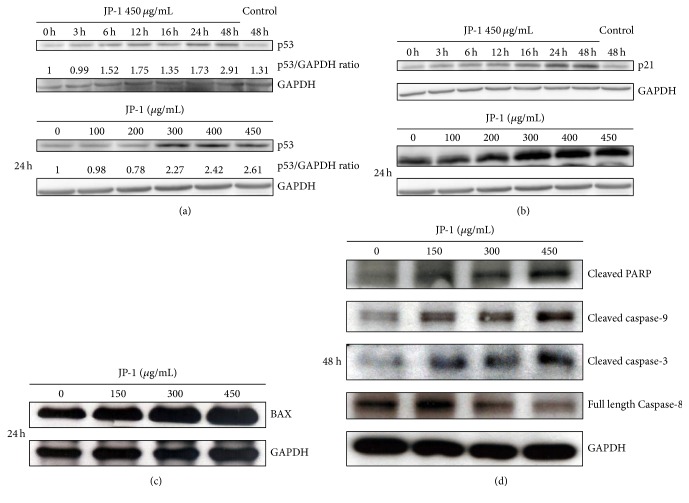
JP-1 induces p53 and its downstream p21 and BAX proteins accompanied by the activation of PARP and caspases in A549 cells. (a) JP-1 increased the p53 protein in a time- and dose-dependent manner. The numbers under the p53 bands show the relative densitometric ratios of p53 to GAPDH bands. (b) Similar time- and dose-dependent increase of p21 protein was observed after treatment with JP-1. (c) JP-1 increased the BAX protein after 24 h of treatment. (d) JP-1 increased the cleaved forms of PARP, caspase-9 and caspase-3 proteins and decreased the full length caspase-8 protein after 48 h of treatment. Cell lysates were analyzed by Western blot, using GAPDH as the loading control.

As AMPK is involved in the activation of p53 [28], an important tumor suppressor and attractive cancer therapeutic target [5], we therefore examined if this powerful transcription factor plays a role in the anticancer activities of JP-1. As expected, the p53 protein level in A549 cells was increased by JP-1 treatment in a time- and dose-dependent manner (Figure 4(a)). Accordingly, its well-known downstream target p21 [15] protein was also induced in a similar manner (Figure 4(b)). In parallel, another p53 downstream, BAX [15], was also increased by JP-1 in a dose-dependent manner (Figure 4(c)). As p21 is a pan CDK inhibitor, which constrains the progression of cell-cycle [15, 29] and BAX can promote the intrinsic/mitochondrial apoptotic pathway [30], the induction of these two proteins was in accordance with the JP-1-mediated growth inhibition and apoptosis induction shown in Figures 1 and 2. In line with this, the increases of cleaved forms of caspase-9, PARP (Poly ADP-ribose polymerase), and caspase-3 and the decrease of full length caspase-8 were observed in JP-1-treated A549 cells (Figure 4(d)). Both the intrinsic (caspase-9) and the extrinsic (caspase-8) apoptosis pathways were activated by JP-1 in A549 cells.

### 3.5. JP-1 Increases the Transcription of miR-34a Accompanied by the Suppression of Its Downstream Targets Required for Proliferation and Apoptosis Resistance in A549 Cells

**Figure 5:**
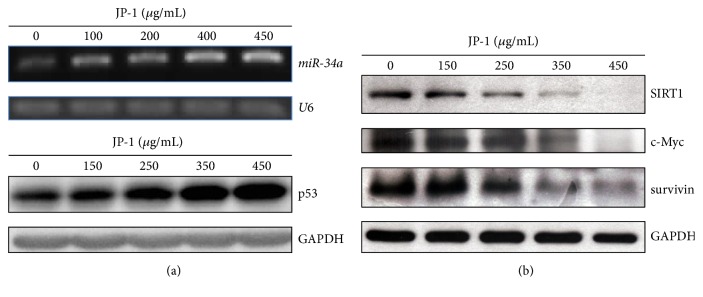
JP-1 induces the transcription of miR-34a and decreases its downstream targets required for proliferation and apoptosis resistance in A549 cells. (a) RT-PCR analysis showed that the transcription of miR-34a was increased along with p53 protein induction (Western blot) after treatment with JP-1 for 48 h. (b) Western blot analysis showed the decreases of miR-34a downstream proteins, SIRT1, c-Myc, and survivin after treatment with JP-1 for 48 h. The U6 small nuclear RNA and GAPDH were used as the loading controls for RT-PCR and Western blot, respectively.

In addition to the p21 and BAX, p53 also is reported to activate the transcription of miR-34a, a potent tumor-suppressive miR, which gains much attention in recent cancer researches [20, 31]. This triggers our interest to investigate the effect of JP-1 on miR-34a transcription. As shown in Figure 5(a), JP-1 dose-dependently increased the transcription of miR-34a in A549 cells along with the induction of p53. As miR-34a has been shown to repress its downstream targets required for cell-cycle progression (c-Myc) and apoptosis resistance (SIRT1 and survivin) [21], we then investigated the changes of these proteins after JP-1 treatment. As expected, significant decreases of SIRT1, c-Myc, and survivin were observed in JP-1-treated A549 cells (Figure 5(b)). The apoptosis induction shown in Figure 2 and Table 1 might be also attributed to the downregulation of these proteins.

### 3.6. JP-1 Downregulates the miR-34a Targets Controlling EMT and Inhibits Migration in A549 Cells

**Figure 6:**
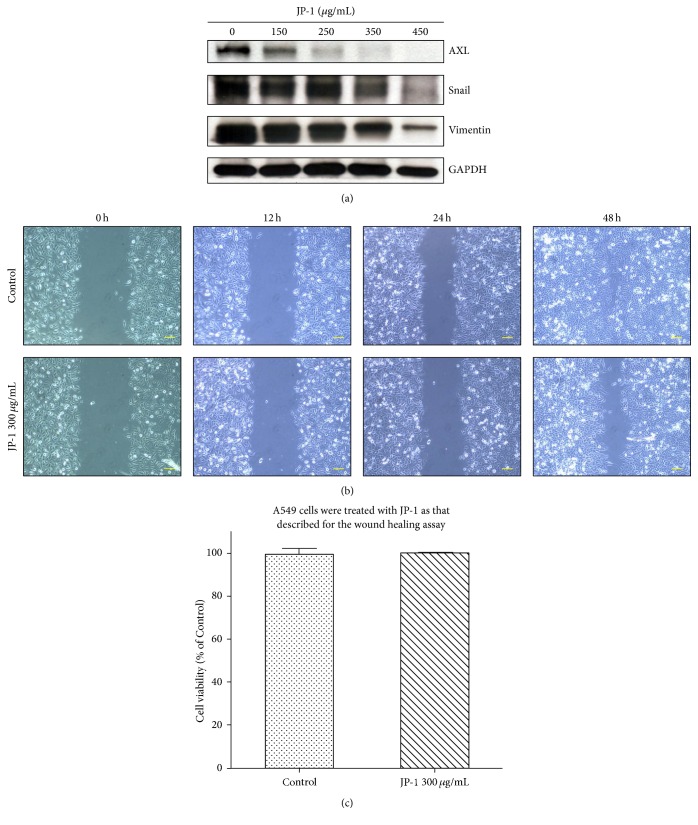
JP-1 decreases the miR-34a downstream targets controlling EMT and inhibits the migration of A549 cells. (a) The decreases of miR-34a downstream EMT inducer (AXL) and marker (Snail) proteins after treatment with JP-1 for 48 h. The downregulation of another EMT marker Vimentin protein was also observed. Cell lysates were analyzed by Western blot, using GAPDH as the loading control. (b) Wound healing assay performed in IBIDI Culture-Inserts. A549 cells treated with JP-1 300 *μ*g/mL or medium only (control) were seeded into different reservoirs of an IBIDI insert as described in Material and Methods. The closure of the gap was photographed by phase-contrast microscopy at 12, 24, and 48 h after treatment, scale bar = 50 *μ*m. (c) The cell viability of A549 cells treated with JP-1 as that described for the wound healing assay. Data are expressed as mean ± SE and analyzed as described in Materials and Methods. At that cell density, JP-1 did not affect the cell viability of A549 cells within 48 h.

MiR-34a also represses the EMT inducer (AXL) [32] and marker (Snail) [33], and this phenomenon has been demonstrated in A549 cells [33, 34]. We thus examined the effect of JP-1 on these two proteins. As expected, JP-1 dose-dependently decreased AXL and Snail proteins after 48 h of treatment (Figure 6(a)). Moreover, it has been shown that repression of Snail by p53/miR-34a axis in A549 cells would result in decrease of Vimentin (EMT marker) expression [33]. In agreement, significant decrease of Vimentin protein level was also found in these JP-1-treated A549 cells (Figure 6(a)). Prompted by the aforementioned results, we examined the effect of JP-1 on the migration of A549 cells. An in vitro wound healing assay was performed using IBIDI culture insert as described in Section 2. Assessment of wound closure 48 h after starting of the experiment revealed markedly inhibited migration of JP-1-treated cells into the cell free gap in comparison to the control (Figure 6(b)), while no growth inhibition effect of JP-1 occurred in this experimental condition (Figure 6(c)).

**Scheme 1:**
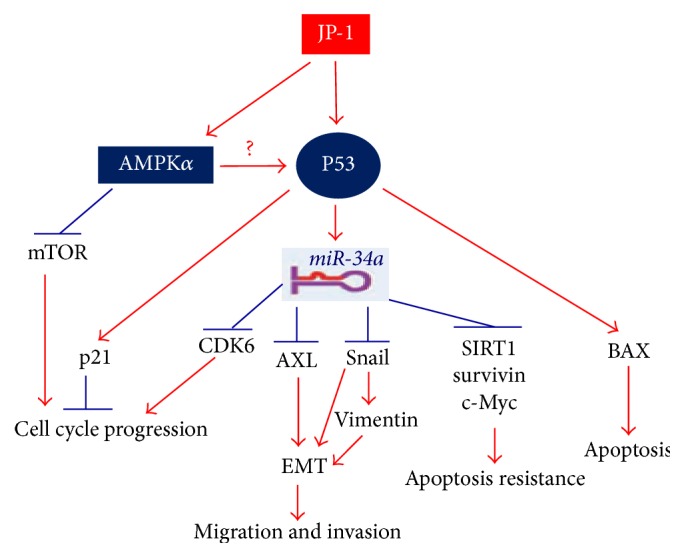
Proposed mechanisms of action for the anticancer activities of JP-1. Through the activation of AMPK and p53/miR-34a axis, JP-1 modulates proteins related to cell-cycle regulation, apoptosis resistance, and EMT.

Collectively, as shown in Scheme 1, the mechanism of action underlying the anticancer activities of JP-1 is proposed mainly through the activation of p53/miR-34a axis to modulate proteins related to cell-cycle regulation, apoptosis induction, and EMT.

## 4. Discussion

The incidence of LADC has increased markedly in the past several decades. It has replaced squamous cell carcinoma as the most prevalent type of lung cancer [35]. In spite of the progress of molecular targeted therapy, the drug resistance develops in most patients and more than 50% of LADC patients still lack targetable mutations, demanding alternative therapeutic approaches [36]. Activation of the tumor suppressor p53 by blocking its negative regulator MDM2 is regarded as an attractive approach for cancer therapy [7]. Preclinical study has demonstrated the anticancer effects of small molecule MDM2 inhibitor both in A549 cells and in patient-derived lung cancer xenograft [37]. Although a variety of MDM2 inhibitors have entered into early phase clinical trials [38], they are still not clinically available nowadays. The toxicity may limit their future clinical use. By contrast, the Chinese herbal medicine often possesses advantages of low toxicity. JP-1 only had mild effect on the growth of HS68 human primary foreskin fibroblast cells as compared to its marked antiproliferation and apoptosis-induction activities in A549 cells. For timely treatment of advanced LADC patients refractory to conventional treatments, the Chinese herbal medicine with activity of restoring p53 level is a potential alternative therapeutic strategy for this demand. In support of this notion, our results demonstrate that the Chinese herbal formula JP-1 used for the complementary treatment of LADC could profoundly upregulate the p53 and its downstream targets in A549 LADC cells during apoptosis induction.

Physiologically, p53 can be activated in response to DNA damage, hypoxia, or oncogenic stress. Besides, there is increasing evidence showing that p53 can also be induced by pharmacological or physiological activation of AMPK (AMP-activated protein kinase), a central cellular energy sensor and metabolic switch, which is activated in response to energy crisis [26, 28, 39, 40]. The activated AMPK promotes phosphorylation and acetylation of p53 to prevent it from being repressed by its negative regulator, leading to cell-cycle arrest or apoptosis of cancer cells [28, 40, 41]. Emerging evidence indicates that cancer is primarily a metabolic disease with disturbances in energy production [42, 43]. Targeting cancer metabolism by AMPK activation has become an attractive strategy for cancer therapy [43, 44]. In JP-1-treated A549 cells, the induction of AMPK*α* phosphorylation was accompanied with significant increase of p53. It is deduced that the activation of AMPK by JP-1 might stabilize p53 through phosphorylation and acetylation, resulting in its accumulation. Further study is warranted to investigate the role of AMPK on the induction of p53 by JP-1.

In addition to the well-known p53 downstream targets such as p21 and BAX, we further unveil the effect of JP-1 on the transcription of miR-34a, which is recently regarded as a novel therapeutic target for LADC [23, 24]. MiR-34a is the first identified and well-studied miR in the p53 regulatory network [20, 31]. Owing to its established role in cancer, synthetic miR-34a mimics are currently in Phase I clinical trials for lung and a variety of types of cancers (NCT01829971) [31]. However, effective delivery of miR-34a mimics to solid tumors is limited by factors, including reticuloendothelial system clearance and nuclease degradation [18]. To overcome this limitation, suitable nanoparticle devices were developed to efficiently encapsulate the miR-34a mimics and protect them from the enzymatic degradation. A lipid/polymer nanoparticle named 7C1 has been employed to deliver miR-34a mimic for treatment of lung tumor in animal model [18]. In parallel, nanoplexes such as chitosan/PLGA (Poly(D,L-lactide-co-glycolide)) and SNALPs (stable nucleic acid lipid particles) have been used for the delivery of miR-34a mimics to treat multiple myeloma xenografts in SCID mice [45, 46]. Regarding the elevated miR-34a transcription and significant repression of its downstream targets in JP-1-treated A549 cells, our results suggest the alternative approach for restoring the miR-34a level in p53 wild-type cancers by treatment with Chinese herbal formula such as JP-1.

Chemotherapy and radiotherapy remain the two main streams of treatment for LADC. It is reported that miR-34a sensitizes A549 cells to cisplatin treatment [47] and in vivo delivery of miR-34a enhances the radiosensitivity of lung tumors through downregulation of the double-strand-break repair protein Rad51 [48]. By increasing the miR-34a transcription, JP-1 might enhance the anticancer effects of cisplatin and radiotherapy.

Another obstacle for successful treatment of cancer is the existence of cancer stem cell (CSC) population. It has been reported that the CD44 (high) tumorigenic subsets in lung cancer biospecimens are enriched for low miR-34a expression [49]. In xenograft tumor model, miR-34a has been shown to negatively regulate the tumorigenic properties of high CD44 expression lung CSCs [22]. In line with these reports, the miR-34a inducer p53 is regarded as the barrier to CSC formation [50]. Compared to normal tissues, A549 cells were shown to possess lower miR-34a level [51]. Similar result was also found by Ma et al. when comparing A549 to the nontumorigenic bronchial epithelium cell line BEAS-2B [52]. The effects of JP-1 on the activation of p53/miR-34a axis shown in this study imply its potential in CSC elimination, which might contribute to the clinical benefit observed in patients.

On the other hand, activation of p53 also modulates the immunity. Previous studies have shown that p53 regulates inflammatory cytokines, toll-like receptors, and IFN signaling and modulates the activation of T-cells and NK cells [53]. By transactivation of miR-34a, p53 was recently shown to repress the expression of programmed cell death-ligand 1 (PD-L1), which is overexpressed in many human cancers, promoting T-cell tolerance and escaping host immunity [53]. Targeting the programmed cell death 1 (PD1)/programmed cell death-ligand 1 (PD-L1) pathway to improve the host immunity is a recent breakthrough in cancer immunotherapy and the clinical benefits have been reported in lung cancer [36]. The repression of PDL1 by miR-34a has been demonstrated in lung cancer cell lines, including A549 [53]. Based on the effects of JP-1 shown in this study, modulation of PDL1 might also be part of the JP-1-activated p53/miR-34a tumor suppressor axis.

According to the foregoing discussion, the potential of JP-1 in the enhancement of conventional cancer therapies, CSC elimination, and immunotherapy deserve in-depth studies and future clinical trials.

## 5. Conclusion

Our results demonstrate that JP-1 activates p53 and its downstream targets such as p21, BAX, and miR-34a in A549 LADC cells. Accordingly, the suppression of miR-34a downstream targets such as CDK6, SIRT1, c-Myc, survivin, Snail, and AXL are observed. This activation of p53/miR-34a axis is proposed to the main mechanism underlying JP-1-mediated anticancer effects. Regarding the recent reported crucial anticancer activities of miR-34a, more comprehensive investigation coupled with clinical trials is warranted for the sake of integrating JP-1 into complementary cancer therapy.

## Figures and Tables

**Table 1:** Cell-cycle phase distribution (%) of 48 h JP-1-treated A549 cells.

Treatment	Cell-cycle distribution			
	Sub-G1 (%)	G0/G1 (%)	S (%)	G2/M (%)
Control	1.9	69.1	13.9	14.2
JP-1 150 *μ*g/mL	2.8	67.9	14.3	13.9
JP-1 300 *μ*g/mL	15.6	56.4	14.0	12.4
JP-1 450 *μ*g/mL	63.8	26.1	6.1	3.7

## Abbreviations

CDK: Cyclin-dependent kinase
GAPDH: Glyceraldehyde-3-phosphate dehydrogenase
LADC: Lung adenocarcinoma
miRs: MicroRNAs
PBS: Phosphate buffered saline
PBST: Phosphate buffered saline with tween 20
SE: Standard error
SRB: Sulforhodamine B.
